# “It was too much for me”: mental load, mothers, and working from home during the COVID-19 pandemic

**DOI:** 10.3389/fpsyg.2023.1208099

**Published:** 2023-10-30

**Authors:** Caitriona Delaney, Alicja Bobek, Sara Clavero

**Affiliations:** AIB Research Centre on Inclusive and Equitable Cultures (RINCE), Technological University Dublin, Dublin, Ireland

**Keywords:** working mothers, COVID-19, working from home, mental load, narrative interviews

## Abstract

This study analyses the experiences of working from home (WfH) during the COVID-19 pandemic and the impact it has on working mothers through the lens of “mental load.” Remote study, often lauded as a way to reduce work/life conflicts, can bring new multifaceted challenges for working mothers and, as this study shows, suddenly shifting to remote work led to the boundaries among work, care, and domestic labour becoming blurred. The data used here are from narrative interviews collected as part of the RESpondIng to outbreakS through co-creaTIve inclusive equality stRatEgies (RESISTIRÉ) Horizon 2020 project, which analyses the impact of COVID-19 policies on gendered inequalities across the EU27 and Türkiye, Serbia, UK, and Iceland. We draw on 12 narratives from working mothers in Austria, Bulgaria, Czechia, Denmark, Ireland, Estonia, Greece, Portugal, Netherlands, the United Kingdom, and Slovakia. Employing thematic analysis, the analysis of these narratives, illuminates the challenges and opportunities of WfH and highlights its impact on mental load. While adding to the research on WfH and working mothers, the analysis also illustrates the lessons to be taken forward as well as underscoring the importance of mental load both theoretically and empirically.

## Introduction

While ‘working from home’ (WfH) had been already adopted as a form of flexible work before the COVID-19 crisis, it became a widespread practice during the pandemic across many European countries as part of public health measures. Working remotely suddenly and unexpectedly due to the COVID-19 pandemic led to the need for many people to adapt to an intense global situation and have to balance work and life in the home space. This study analyses the experiences of WfH and the impact it has on working mothers through the lens of “mental load,” understood as a combination of cognitive labour, physical labour, and emotional labour (Dean et al., 2022).

The abrupt transition to WfH led to work and family colliding for a lot of people, thus resulting in many working mothers conducting a ‘split shift’ during the pandemic (Pettigrew, 2021, p. 8). Examples of a split shift include getting up earlier than children to do paid labour, then moving on to domestic and care labour, and returning to paid labour in the evening (Garner, 2020). Quite importantly, due to the sudden and unique nature of this recent shift, WfH during the COVID-19 pandemic led to home becoming an “ambiguous space” (Boncori, 2020, p. 6), with home no longer being a space to retreat from the world of work (Hartig et al., 2007). Instead, for many individuals, WfH resulted in a feeling that one’s home was ‘invaded’ by work obligations and increased unpaid labour such as emotional labour (regulating one’s own and other people’s emotions) (Hochschild, 1983) and cognitive labour (regulating one’s own emotions and/or colleagues and family members) (Daminger, 2019), in addition to increased domestic duties (Boncori, 2020).

Against this background, this study explores the experiences of working mothers who WfH during the pandemic, with the aim to answer the following research questions: What were the experiences of mothers who worked from home during the COVID-19 pandemic? Did working from home during the pandemic impact the mental load carried by them? What pandemic lessons can we learn from the data as people increasingly work from home? These research questions are in line with the ambition of this study to move toward a holistic understanding of mental load and working mothers and to increase the visibility of unseen labour by exposing how working mothers experience mental load and how this impacts their everyday lives.

Methodologically, the study draws on narrative interviews conducted with working mothers to discuss their experiences of the pandemic. We explore how a heavy mental load, encumbered by personal and professional obligations, affects their professional opportunities and their personal wellbeing. As such, this study contributes to the existing literature on mental load, advancing existing knowledge on this issue, which is conceptually crucial for understanding the unseen aspects of work for mothers. Our analysis demonstrates that carrying a heavy mental load impacts work–life balance and wellbeing. While the narratives used for this analysis refer specifically to the context of the pandemic, they provide examples of the challenges and opportunities that many mothers in paid employment may encounter post-pandemic in the ever-changing world of work.

The data used in this study were gathered as part of the RESISTIRÉ project, a research and innovation project, funded under EU Horizon 2020 which aims to reduce gender+ inequalities caused by policy and societal responses to COVID-19. An intersectional gender+ approach highlights gender relations and gender inequalities and acknowledges the mutual shaping of multiple complex inequalities, recognising the intersection of gender with age, race/ethnicity, class, disability, and sexuality (Walby et al., 2012; Verloo, 2013). We address these issues in the discussion section.

Sections one and two provide the conceptual framework and background context for this study, by critically reviewing the literature on mental load and (WfH) during the pandemic. Section three outlines the methodological approach underpinning the study with specific recourse to narratives and thematic analysis. Moving to the fourth section, the results of the analysis are presented and discussed. This study finishes with a concluding section that summarises the main findings and outlines the closing arguments.

## Mental load

Operating at both the familial level and the societal level, the mental load has three characteristics. First, it is carried out internally, it is invisible, and it “results in a range of unpaid, physical labour” (Dean et al., 2022, p. 13). Second, it can permeate all spheres of an individual’s life. Third, it is never-ending and unbounded, which is due to its emotional element (Dean et al., 2022, p. 13).

Mental load involves both emotional labour and cognitive labour, in addition to physical labour. Emotional labour, generally conducted by women and often invisible, includes the regulation of one’s own and other people’s emotions (Hochschild, 1983). Frequently conducted within family units, emotional labour involves providing emotional support to family members and often happens in conjunction with physical labour (Curran et al., 2015). Many women feel the need and desire to conduct emotional labour. However, tensions may arise when women feel that they are expected to carry out emotional labour in conjunction with or at the detriment of being able to pursue their personal goals (O’Brien, 2007). Emotional labour has a particularly negative impact on mothers who are marginalised, who struggle financially, and who have little emotional support (O’Brien, 2007). During the pandemic, the emotional labour conducted by women was greater than pre-pandemic, which is partly attributable to COVID-19 adding to the expectation felt by many working mothers to keep their families safe and calm (Pettigrew, 2021). Moreover, intensified instances of emotional labour during the crisis were often accompanied by an emotional cost (Hjálmsdóttir and Bjarnadóttir, 2021), which led to increased levels of burnout (Thompson, 2022). The emotional element of mental load can be understood as being ‘emotional’ because the mental load is underpinned by wanting to ensure that one’s family unit has “a positive emotional experience […] which inevitably means managing one’s own and the family’s emotions*”* (Chung et al., 2021, p. 7).

The mental load is also comprised of cognitive labour, which refers to the “(1) work of anticipating needs; (2) identifying options for meeting those needs; (3) deciding among the options; and (4) monitoring the result” (Daminger, 2019, p. 610) to ensure family units work smoothly. As it is invisible, cognitive labour is particularly stressful when it is not recognised as actual work (Nomaguchi and Milkie, 2020, p. 207). Due to cognitive labour taking place internally, thus rendering it invisible, it is hardly surprising that it is problematic to measure. However, existing indicators suggest that cognitive labour is “highly gendered, with women often serving as household managers who delegate tasks to their male ‘helpers’” (Daminger, 2020, p. 808). Furthermore, it is mothers who tend to do the larger share of cognitive labour (Nomaguchi and Milkie, 2020, p. 207). Arguably, the COVID-19 pandemic led to stress, pressure, and “the unequal gender distribution of cognitive labour” (Czymara et al., 2020, p. 11). It has been suggested that this may be connected to wider issues concerning greater possibilities for “deteriorating career prospects for women than for men and the widening of the gender wage gap during the recovery process” (Czymara et al., 2020, p. 11). Additionally, the pandemic appears to have increased the “existing differences at the cognitive level of work further*”*; for example, working mothers tended to be more concerned about childcare as opposed to fathers being more focused on their paid labour (Czymara et al., 2020, p. 11–12). Moreover, cognitive labour is often a source of relationship conflict and may also be a distraction from paid work and leisure activities (Daminger, 2019).

It is the stress and strain of conducting cognitive labour, physical labour, and emotional labour that leads to labour becoming a mental load. As a theoretical lens, mental load allows for a rounded conceptualisation of the load carried by working mothers in their daily lives due to combing paid labour with unpaid labour, seen and unseen tasks.

The way mental load operates specifically, never-ending and unbounded, results in the boundaries between paid and unpaid labour, as well as among down time, paid labour, and family obligations, becoming blurred (Chung et al., 2021, p. 7). Arguably, it is women who disproportionately carry the mental load within family units (Fielding-Singh and Cooper, 2022), with family being the main recipient and location of this unpaid labour (Dean et al., 2022). During the pandemic, many women and mothers experienced, “a persistent elevated mental load […] from familial, professional, and social networks” (Pettigrew, 2021, p. 8). A heavy mental load has widespread consequences, particularly for working mothers who do not have strong support networks. The consequences may include negative impacts on one’s emotional, physical, and economic wellbeing and concrete obvious impacts such as carrying a heavy domestic load (Dean et al., 2022). As Ruppanner et al. (2021) have shown, a heavy mental load because of managing one’s household impacts working mothers’ ability (e.g., time and mental space) to conduct paid labour. Indeed, both the physical act of conducting domestic labour and the cognitive and emotional aspects need to be accounted for to enable a rounded and full understanding of the impact of paid and unpaid labour on inequality in the home and to allow for realistic ways to address labour inequality in the domestic sphere (Chung et al., 2021).

## Blurring work–life boundaries during the pandemic

WfH was an issue that affected many individuals during the pandemic and will continue to have an impact in the post-pandemic world. WfH may benefit both employees and employers by improving job satisfaction and reducing work/family conflict. However, a sudden shift to WfH, accompanied as it was by “increased job and domestic demands and reduced support [during] the COVID-19 pandemic, disrupted the work–nonwork boundary for many workers” (Schieman and Badawy, 2020 in Kossek et al., 2021, p. 1616).

It needs to be emphasised that many individuals had experienced WfH and/or hybrid work before COVID-19 restrictions were introduced (Eurofound, 2022). While some argue that WfH potentially reduces work/family conflicts for working mothers, the reality often entails more childcare and domestic responsibilities, with paid labour receiving less attention (Carli, 2020). This increase in unpaid labour (childcare and domestic labour) is arguably connected with working mothers often prioritising unpaid care and domestic labour over paid labour (Lilly et al., 2007). Indeed, there is a wealth of data and research showing that, before the pandemic, working mothers spent substantially more time on unpaid domestic labour than men (e.g., Craig and Mullan, 2011; Bianchi et al., 2012; Kuhhirt, 2012; Raley et al., 2012; Evertsson, 2014; Argyrous et al., 2017; Sullivan et al., 2018; McMunn et al., 2020).

This was intensified during the pandemic (Hess et al., 2020) which in many ways may be attributable to informal networks’ support (family and friends) being removed because of public measures that limited people’s ability to meet as well as the removal of more formal supports such as childcare services (Fodor et al., 2021). The sudden closures of schools and childcare facilities increased the care burden on working parents (Yerkes et al., 2022). While parental financial status and time availability mediated the experiences of mothers and fathers during the pandemic (e.g., Carlson et al., 2020), studies have shown that this had a particular impact on mothers and thus limited the time they had available to spend on paid labour (Power, 2020; United Nations, 2020). Studies have found that mothers often encountered additional stressors and increased workloads due to WfH amidst school closures (Darmody et al., 2020; Moore et al., 2020; Reichelt et al., 2020), while fathers were inclined to engage more readily in ‘high reward’ activities such as fun activities and supervising children (Clark et al., 2021). Women’s psychological and emotional wellbeing was negatively impacted due to changing and intensifying family dynamics during lockdowns (Clark et al., 2021). The pandemic arguably both revealed and reinforced gender norms and gendered expectations particularly for women who are also mothers, regardless of them being in dual-income partnerships or not (Hjálmsdóttir and Bjarnadóttir, 2021). Moreover, working mothers were more likely to reduce or stop their paid labour, and even women in dual-working couples tended to invest more time in childcare than men (Alon et al., 2020; Andrew et al., 2020; Fodor et al., 2021). Women were particularly burdened during COVID-19 because of balancing paid and unpaid work such as physical, emotional, and cognitive labour (Collins, 2020; Collins et al., 2020; Thibaut and van Wijngaarden-Cremers, 2020; Hjálmsdóttir and Bjarnadóttir, 2021). Indeed, WfH is one way in which gender inequality may be perpetuated because it is usually women who conduct both paid labour and domestic labour (Sullivan and Lewis, 2001).

Overall, research conducted during the pandemic has shown a diverse picture of the consequences of suddenly having to work from home for working mothers. For some working mothers, the shift in boundaries between paid and unpaid labour led to reduced work–life balance due to the amplification of work pressures (Yerkes et al., 2022). Conversely, some working mothers reported more support from their husbands/partners as a positive ramification of COVID-19-related changes to how and where people worked (Yerkes et al., 2022). Other important factors affecting how working mothers experienced WfH during the pandemic in relation to their work–life balance include the age of their children, family status, whether they worked full or part time, and the work–care regime in their respective countries (Yerkes et al., 2022). The difference in experiences may be attributed to the different political and social contexts and varying work–family policies in countries before and during COVID-19 (Koslowski et al., 2022), such as policies that aim to facilitate combining paid work and care work, reduce gender inequalities, improve levels of paternal participation with care work, reduce poverty levels, and improve outcomes for children both educationally and socially (Yerkes and den Dulk, 2015; Javornik and Kurowska, 2017; Nieuwenhuis and Maldonado, 2018; Pavolini and Van Lancker, 2018). The variations in these policies have consequences for the support or lack thereof for working mothers in relation to childcare, etc (Leitner, 2003; Lohmann and Zagel, 2016).

However, despite work–family policies varying across different state contexts, working mothers in a myriad of social contexts often are expected to ‘do it all’, with the implication that it is them who carry the brunt of domestic responsibilities as well as working full time (Knijn and Kremer, 1997; Wierda-Boer et al., 2009; Knijn and Da Roit, 2014; Yerkes and Hewitt, 2019). Indeed, working mothers ‘down time’ often involves ‘playing catch up’ on care and domestic duties, with leisure time becoming contaminated and fragmented (Mattingly and Bianchi, 2003). This can in turn negatively impact how working mothers perceive their work–life balance as they feel permanently under time pressure (Gimenez-Nadal and Sevilla-Sanz, 2011). Despite WfH/hybrid work being often lauded as the great fix for working mothers (e.g., improving work–life balance) during the pandemic, those who worked from an external workplace were better able to combine their paid and care work than those who WfH (Yerkes et al., 2022).

The rapid changes resulting from the pandemic impacted working mothers with young children in many significant ways. First, as evidence shows, they reduced the hours they spent in paid labour (Collins et al., 2020; Myers et al., 2020). Second, there is also strong evidence that family obligations and expectations around domestic labour primarily fell on women, following a long-established pattern of women taking on caring and unpaid domestic labour and carrying out more unpaid caring work than men (O’Brien, 2007; Craig and Churchill, 2020). Third, home as a space to retreat from the world risked being colonised by becoming a workspace, working mothers then had to conduct paid and unpaid labour in the same space with no opportunity for ‘escape’ from work and/or care obligations (Byrne, 2020).

It has been argued that a need to ‘do everything’ is connected with constructs of ‘ideal motherhood’ and ‘intensive mothering’ which influence the impetus felt by many working mothers to engage in ‘maternal gatekeeping’ (Gaunt, 2008; Puhlman and Pasley, 2013; O’Sullivan et al., 2022). ‘Maternal gatekeeping’ is underpinned by anxiety by working mothers about social judgement should they fall short in meeting the ideals regarding care and domestic responsibilities (Gaunt, 2008; Puhlman and Pasley, 2013). The pressure to be an ‘ideal mother’ or a ‘perfect mother’ is connected with elevated feelings of guilt, burnout, strain, and anxiety and may lead to some working mothers reducing their paid labour and career ambitions (Roskam et al., 2017; Meeussen and Van Laar, 2018). Indeed, maternal gatekeeping, associated as it is with internalised notions of being a ‘perfect mother’, often leads to mothers ‘guarding’ domestic and care tasks (Meeussen and Van Laar, 2018), which in turn leads to working mothers doing more care and domestic tasks. This adds to their workload, both paid and unpaid, subsequently increasing the second shift—which refers to the unpaid work conducted by in-the-home women after they have done paid labour—generally outside the home (Hochschild and Machung, 2012).

The idea of the perfect mother is connected with the deeply ingrained idea that motherhood comes naturally and easily to all women (Staneva and Wittkowski, 2013). Having it all, a wonderful career, happy children, and a clean house, is part of the perfect mother performance, and despite critique of this construct by many mothers, the ideal prevails and pressures many mothers contemporarily (Staneva and Wittkowski, 2013). The cost of attempting to be a ‘perfect mother’ goes beyond the personal as it can result in gender inequalities being reinforced at both the macro and micro levels (e.g., working mothers being underrepresented at higher levels of the labour market, lower labour market participation, and lower wages) (European Union, 2016).

In the following sections, our analysis shows the extent to which WfH may lead to blurred work–life boundaries and increase the mental load carried by working mothers alongside their paid labour.

## Methods

### Participants and procedure

The data in this study were collected as part of the RESISTIRÉ project via individual narrative interviews to gather information on people’s lived experiences of both the pandemic and the policy responses to COVID-19. The narratives were conducted across the EU27 (excluding Malta), Serbia, England, Iceland, and Türkiye by 31 National Researchers (NRs) in two different cycles. This study is based on the data collected during the first two cycles. A total of 188 narrative interviews were conducted in cycle 1 (August 2021) and 306 in cycle 2 (February 2022). The narrative interviews started with a general background question followed by an open question: Many persons have been affected by the COVID-19 situation in different ways. Can you describe to me how you have been affected by COVID-19 and what this has meant for your situation? The goal of the interviews was for the narrator to remain as the central actor throughout with the interviewer having the role of an ‘active listener’. They could occasionally ask clarifying questions but did not interfere too much with the story being told. To verify that they had understood the narrators correctly, the interviewer ended the interview by retelling the story back to the narrator, thereby allowing the narrator to correct any misunderstandings. The interviews lasted 1 h (approximately).

The narratives that were not conducted in English were translated by the national researchers. Participants were recruited via consortium partners’ existing networks, snowballing, and online searches. The narrative interview technique “entails a conceptual shift away from the idea that informants have answers to questions posed by an interviewer, and toward the idea that informants are narrators with stories to tell and in their own voices” (Axelson et al., 2021, p. 12; Chase, 2005; Kim, 2016).

All the narratives from both cycles (424) were imported into NVivo where they were assigned attributes based on the policy domains chosen by the NRs. The policy domains are the gender equality domains of the European Commission Gender Equality Strategy 2020–2025 (European Commission, 2020) and the Beijing Platform for Action (United Nations, 1995): work and the labour market, care, pay and pension, decision-making and politics, gender-based violence, fundamental human rights, economy, and environmental justice. Using the attribute function to select narratives relevant to a specific policy domain, the narratives were scrutinised again, one domain at a time. In the final stage of the analysis process, when the results for each domain were summarised, specific attention was paid to the salience of different inequality grounds.

The narratives used in this study were specifically selected based on the respondents’ profiles. As the domain of work is of specific interest for the present study, narratives pertaining to this domain, specifically those on WfH during the pandemic, were scrutinised by the lead author of this study and selected using criteria such as gender, parental status, employment status, and place of work during the pandemic. When specific narratives were chosen and deemed the most illustrative of how WfH during the pandemic impacted the mental load of working mothers, then a thematic analysis (Braun and Clarke, 2006) was conducted on these specific narratives. The narratives chosen were (1) particularly illustrative of how working mothers experienced balancing their paid and unpaid labour and how this led to increased stress, reduced wellbeing, and an overall increase in the weight of their mental load and (2) provided strong examples of the impact of WfH on working mothers during the pandemic, therefore providing supporting evidence to previous studies. In this selection process, the lead author made the initial database search and analysis while the co-authors read and cross-checked the narratives to ensure rigour. The three authors coded and took part in the thematic analysis to ensure the process was thorough and that the themes were salient.

The authors of this study are aware that the experiences presented in the results section are not exclusive to the narrators included in the analysis. Rather, the narratives analysed here illustrate how these particular working mothers experienced increases in their mental load due to the sudden switch to working from home due to COVID-19. Comparing these respondents’ experiences of WfH during the pandemic with those of respondents with different profiles could enhance the solidity of results (e.g., women WfH without children), and that strategy was deemed outside the scope of this study.

Narratives from Austria, Bulgaria, Czechia, Denmark, Estonia, Greece, Ireland, Northern Ireland, Netherlands, Portugal, the United Kingdom, and Slovakia were selected against these criteria. From the 12 narratives selected, all of the respondents were working mothers who usually worked outside of their homes. Four of the women parented alone, while the other eight women had partners—all of them men. Seven of the women were in their 40s (from 40 to 48 years old), two of them were in their 30s (35 and 36), and one was 29. One woman was referred to as middle-aged in the original narrative and one woman did not give her age. Most of the women interviewed had school-age children ranging from junior to senior school, some had children who were young adults, and one woman had a new-born baby.

### Ethics statement

Informed consent was obtained from all participants. In the cases where the interview was conducted online, consent was given verbally and recorded. All the data were anonymised, and all names used in the findings section are pseudonyms. RESISTIRE partners involved in data collection submitted their individual research plans to their institutions’ ethics committee for review and approval. Consortium partners abided by the national legislation requirements in their national jurisdiction.

### Data analysis

Mental load is the theoretical lens through which working mothers’ experiences of WfH during the pandemic are explored, to take lessons from these experiences into the post-pandemic world of work. As such, the overarching theme is ‘mental load’, although overload and coping are key concepts that also emerged from data analysis. Within this overarching theme, care, support, and space emerged as key salient themes within the narratives. This thematic analysis process was guided by Braun and Clarke’s (2006) six-phase approach. Their approach includes identifying patterns and/or themes in the data. The analysis began with data familiarisation, generating initial codes (coding for this study was conducted by the lead author with the interrater agreement being reached with the co-authors), reviewing and then defining themes, and ending with the writing-up phase (Braun and Clarke, 2006). The lead author initiated this process with the co-authors independently reading the narratives while being cognisant of the research questions (Jones et al., 2011). The analysis process was not linear as the analysis involved moving between the six phases outlined by Braun and Clarke (2006) (Maguire and Delahunt, 2017). Guided by the specific research questions outlined earlier in the study, our analysis was driven by an analytic focus on working mothers’ experiences of WfH while attending to unpaid labour.

Mental load emerged inductively from the data as theoretically significant for the study and subthemes were also identified (Table 1). The subthemes of care are care and work, childcare, and self-care, while the subthemes of support are family support and employer support. Finally, the subthemes of space are overburdened, blurred boundaries, mental space, physical space, split shift, and never-ending shift. It must be noted that many of the themes intersect, such as care and support, care and family status, support and family status (e.g., being married, single, or parenting alone), and space and self-care. These themes and subthemes highlight and reflect the mental load carried by the respondents.

**Table 1:** Analytical themes, themes intersections and subthemes.

Theme	Subtheme	Intersections
Care	Care and work	Care and support
	Childcare	Care and family status
	Self-care	Care and work and space
Support	Family support	Support and family status
	Employer support	Support and care (of others and self)
Space	Overburdened	Space and self-care
	Blurred boundaries	Split shift/never-ending shift

## Results

Below we discuss the main themes that emerged inductively from the data.

### Care and work during the COVID-19 pandemic

The respondents’ narratives illustrate the myriad ways in which WfH in conjunction with conducting care work added to their mental load during the COVID-19 crisis. Respondents frequently talked about how caring for children during the pandemic, home schooling, and conducting their own paid work was a challenge that added to their mental load. The extra care work included intensified emotional and cognitive labour as the extracts below illustrate. Combining care with paid work was, for example, a particular challenge for Olga, a 40-year-old woman living in Estonia with her partner and three children (a baby and two primary school-age children). In the interview, Olga describes combining care tasks with cognitive labour and how these experiences contributed to a heavy mental load. Olga outlines the planning (cognitive labour) she conducted and how she needed to work a ‘split shift’ to enable her to attend both her paid and unpaid labour obligations. Specifically, Olga carried out her paid labour at night when her unpaid work had finished for that day. Below, Olga talks about WfH:

The role division is clear. I have to be mother first and foremost […] Pandemic or not, meals have to be cooked, laundry done and ironed […] the brunt falls on me […] The times when I was able to do my own work were very limited, some hours when my daughter was asleep or I can read something when I am putting her to bed […] There were times when I fell asleep myself when putting her to bed and woke up at midnight to do my work for a few hours in peace and quiet (Olga).

The lived reality of combining care work and paid work as outlined by Olga above was a recurring theme across many of the narratives. For example, Maria, a 45-year-old mother living in Bulgaria with her partner and two children (7- and 12-year-old girls), talked about being a “girl for everything.” This sentiment was echoed by many working mothers across the narratives. Maria elaborates on what doing ‘everything’ meant for her:

There was work for three mothers at home, and I was only one. I had to be a mother, a teacher, a partner, a doctor - all from the same room at home. I had a lot of worries […] At the same time, my daughters needed more care - private lessons, therapy, and what not. I was getting very anxious about my financial situation […] As a whole, I had to completely ignore my personal needs during the pandemic. There was so much tension in me and at home (Maria).

Maria’s narrative above highlights how her cognitive labour—for example, organising her various roles and tasks in combination with the increased emotional labour she conducted for her daughters—collided, resulting in conflicting demands on Maria’s time which in turn limited the time and space available to her for self-care. All of the obligations and emotional and physical drains on Maria added in turn to the weight of her mental load. For Maria, doing paid labour and caring physically and emotionally for her children in the same space overburdened her, thus, illustrating the intersection of care and work and space.

The experience of care and work during the pandemic is further outlined by Bethany, a self-employed, married, 29-year-old mother of two (7 years old and a new-born baby) from the United Kingdom:

This was a very stressful time, childcare and work were lumped together due to the pandemic. […] I did not know whether my business would collapse if I took leave, I wasn’t sure whether my staff would be able to continue without me […] My husband was really busy with work since construction sites could stay open at first, which meant I had to take on everything at home myself. I had my son in a baby carrier while I was in back-to-back zoom meetings trying to work out how to keep the business afloat and researching what support might be available. It was an absolute mess! We stopped home schooling my daughter for a while because it was impossible to keep up with everything […] it was a lot to think about all at once, especially since a 7-year-old and a new-born have such different needs (Bethany).

As outlined above, these working mothers experienced increased pressure and a heavier-than-normal mental load due to WfH during the pandemic. For many of the respondents, care and domestic labour took precedence over paid labour. In addition, regardless of having a partner or not, the narrative extracts illustrate that these working mothers took on the bulk of care and domestic labour while also conducting paid labour. Moreover, the narrative extracts in the preceding section demonstrate the multiple ways in which care work in conjunction with paid labour impacted the respondents’ mental load. The extracts also illustrate that the issues these working mothers experienced are multifaceted and how care and paid labour interact.

### Support

As previously stated, several of the themes intersect. The intersection of support and family status and the intersection of support and care (both of others and self-care) point toward how dynamic mental load is, the ‘messiness’ of WfH, and how mental load interacts with life and obligation—specifically care and domestic responsibilities. Beginning with the intersection of support and family status from the perspective of working mothers parenting alone, we draw below from Magda’s narrative. This narrative illustrates how parenting alone while WfH impacted Magda’s mental load and how the absence or presence of support contributes to the weight of the mental load carried.

Magda is 43 years old; she lives in Ireland with her son (8 years old) and is originally from Poland. This narrative shows the practical and emotional challenges Magda faced due to WfH with a young child during the pandemic. Magda outlines how:

When you work in the office, and then you all the sudden work remotely, and you are at home with a kid, it was hard to get the work done. And at work in general, it was a disaster, because nobody was prepared for the remote work. So, people did not know what to do (Magda).

Magda also spoke about the guilt she felt because she did not have enough time for her son:

I remember that we were crying together […] because I felt so bad about the fact that I never had time for him that there was always something else I had to do (Magda).

The collision of care work and paid work and the subsequent ramifications of this collision on Magda’s mental load are visible in her narrative. Similarly, Madalena, a 44-year-old from Greece with a 7-year-old son, reflected on how parenting alone contributed to her heavy mental load and how this impacted her life. Commenting that single parents were ‘forgotten’ during the pandemic, Madalena also mentioned that having a supportive employer and flexible paid work is helping her to balance her obligations. Thus, Madalena’s narrative illustrates the subtheme of employer support and the intersection of support and family status:

My work schedule was completely transformed. I could not concentrate when I was at home because my son needed attention […] His father did not help us at all […] It was a very unjust division of labour. My personal life has diminished […] I had flexible working hours and could handle things. But if I did not, if I was working fixed hours, it would not have worked (Madalena).

Earlier in her narrative, Madalena talked about going to stay with her mother in the countryside and how this support eased her mental load. In addition, Madelana mentions flexible work and she talked about having a supportive employer who in ways helped her to negate the lack of support she talked about receiving from her sons’ father, thus suggesting the importance of support and how support interacts with family status.

The intersection of support and family and its impact on mental load and burnout are highlighted in Diana’s narrative below. A 44-year-old working mother living in Denmark with her two children (6 and 13 years old), Diana, talked about feeling alone whilst balancing paid labour with emotional and cognitive labour which often meant prioritising her children’s needs above her own. The following quote illustrates how Diana felt overburdened due to feeling she was unable to deal with her own paid labour and care obligations:

I was max pressured in terms of being enough in all areas […] it has been hard. At one point I completely lost motivation. I sort of felt that nothing mattered, because I wasn’t doing enough anywhere (Diana).

The loss of motivation mentioned by Diana points toward feeling burnt out. Furthermore, Diana talked about having a supportive employer who allowed her to work from the office for a few days a week when WfH, and how home schooling her children began to severely impact her mental load. Diana recalled how she contacted her manager to discuss how overburdened she was feeling and that this led to her being:

allowed to go to the office […] It helped [because] I sort of had all of the roles […] In one way I had to be the loving caregiver. In another way I had to be the person to say that they had to sit quietly […] There was no one where I could say: ‘You have to take over here’ […] It became a vicious circle […] With my employer I felt, I will do what I have the time to do, but with my children I felt insufficient (Diana).

Parenting alone and WfH as described by the respondents above shows that support from familial networks and/or employers is important to one’s experience of mental load and congruent feelings of overwhelm and burnout. Specifically, Magda did not have support largely due to the lack of familial and social support networks, whereas Madelana had sporadic family support and a supportive employer who reduced her mental load at times, thus highlighting the intersection of support and family status and the difference support makes to an individual’s mental load. Equally, the lack of support Diana had made her mental load very heavy which was mediated by a supportive employer.

Married working mothers of children who were supported also struggled with combining care work and paid work during the pandemic as illustrated below by Doris, a 48-year-old married mother of three children aged 7, 12, and 15, living in Austria.

I cannot exclude emotions when talking about what had happened. My husband and I took turns, one of us was responsible for home-schooling and looking after the children, while the other person was working. But the workload of home-schooling, reproductive work and [my] job was intolerable for me (Doris).

The extracts above suggest that the impact of having to suddenly WfH during the pandemic and having to balance this with care and domestic obligations was ‘intolerable’, as Doris noted. Another example of a married working mother whose mental load was adversely impacted and who encountered negative career ramifications is Marta, who is 35 years old and lives in Czechia with one son who is 4 years old. Marta talked about the impact of formal and informal childcare (grandparent support) being removed during the pandemic:

We were left with no babysitting […] my husband and I were WfH, balancing our work obligations and taking care of our son. But since he is the one who is in a more senior position and earns significantly more money, it was usually me who dealt with the caring responsibilities […] I had an intense sense of hopelessness with no end in sight. […] I managed to fulfil my essential work-related duties like teaching or grading, but I could not work on other things […] My work life got really affected […] the government needs to focus on the domain of care more to create such conditions for parents to be able to work […] because working and taking care of children full-time is in the long run unsustainable (Marta).

Marta’s narrative highlights the pressure that resulted from the removal of formal childcare—creche services and informal childcare support that had been provided by her child’s grandparents. The impact on her paid labour is also illustrated, namely how due to wage discrepancies she had to do most of the childcare which meant she could not give as much time to her paid work. Among the challenges discussed by the respondents were the issue of space, the difficulties of working in one’s home space, and how conducting paid and unpaid labour in the home led to a heavy mental load, to which the study now moves to.

### Space, working from home, and blurred boundaries

Paid and unpaid labour happening in the same space, the home space, led to many respondents feeling overburdened as their homes, generally a space of retreat from the world, became both their workplace and their family space. Emotional labour performed was often intensified due to the pandemic, with the boundaries between work and home becoming porous. Mental load often increased because both paid and unpaid labour happened in the same place—the home. For example, Oonagh, a divorced mother of two older children (12 and 20 years old) and a part-time care worker in sheltered accommodation in Northern Ireland found that having to make regular difficult phone calls to clients as part of her paid work added to her mental load. Providing emotional support to the vulnerable group she works with left Oonagh feeling exhausted emotionally. In her narrative, the impact of performing work-related emotional labour while WfH is visible. The following extract from her narrative illustrates the blurred spatial and emotional boundaries that impacted Oonagh’s mental load and also highlights the impact of feeling unsupported upon Oonagh:

It was too much for me, listening to this all the time. I was a shoulder to cry on, 36 tenants I was phoning every day, including holidays, but I had no shoulders. I felt so exhausted (Oonagh).

Lisa, who is 36 years old and has a daughter who is 8 years old, lives in the Netherlands with her parents alone. Lisa’s narrative illustrates the consequences of living in a small space on her mental load and similar to Oonagh’s narrative above talks about how blurred boundaries between home and work also impacted the weight of her mental load. Lisa noted her employers had a flexible attitude toward employees with children during the pandemic but that this attitude waned over time:

It became normal to be available 24/7 […] it was hard to cook dinner around seven pm but also be available at work […] I was hit by the pandemic, namely mentally. The pandemic was an attack on my private life. I had no feeling of “switching off” […] living in a small house did not help, because my living room became my workplace […] I also had to become ‘a teacher’, it became too much. […] This led to multiple mental breakdowns for me at home. The longer it lasted, the harder it became […] work and caring for [my daughter] put a lot of pressure on me. The increasing pressure resulted in my request at work to be unavailable from 10 am till 12 am. As I had to assist my daughter between these hours in her online call for school, I could not be available at work as well. This is a situation with a lot of frustration. […] My private life was put on hold […] Another difficulty I experienced is the contact I have with my ex-husband. I cannot count on him or find support with him […] my work-life balance became one big spaghetti ball […] That is why I decided to start working at the office for two half and days a week. This was necessary for my mental state (Lisa).

For Lisa, a flexible employer and the support from her colleagues (through daily check-in phone calls, for example) helped her to navigate the extreme pressure she felt due to balancing her paid and unpaid labour obligations in her small home. While for Filipa, a 44-year-old mother of two from Portugal, the blurred boundaries between home and work led her to feel overwhelmed:

I remember being in the kitchen in an online meeting at nine pm and had to pull my stuff aside for them to pass to go to the fridge and prepare dinner. The three looking at me with a furious look feeling their home invaded, that was difficult […] In the first confinement, I had everything organised at home, in the second I had not. In January 2021, I passed a phase I cried all the time. […] I think I got depressed and having things disorganized at home did not help (Filipa).

Jana a married woman with school-going children (age and number of children undisclosed) from Slovakia felt immensely pressurised from conducting her paid work and having to attend to her unpaid domestic duties in the same space. This pressure was compounded by sharing physical space with her family and having to work from her kitchen:

I spent all the day in the kitchen, teaching, cooking, being with my family and a dog was there, sometimes even two […] I mean, others they had their offices in their rooms and for them, the kitchen was still a kitchen, a family place. For me, it was the only place during the day [that] I worked, cooked [and] worked again [in]. The boundaries were blurred. It was really exhausting, sometimes I felt trapped in my kitchen (Jana).

This section has shown the myriad ways people experienced WfH during the pandemic. Care work and domestic labour often took precedence over paid labour with self-care getting regulated to the bottom of a very long ‘to do’ list. Thus, the working mothers’ narratives in this study suggest that the pandemic was a period during which they carried a particularly heavy mental load, resulting in stress, burnout, and an overall reduced sense of wellbeing.

## Discussion

The analysis of the narrative interviews illustrates the multi-layered experiences of and the relationship between balancing paid and unpaid labour and mental load carried by working mothers during the recent COVID-19 global pandemic. The narratives highlight how for many women their mental load became overwhelming because of WfH and changes in the amount of the often-unseen labour they conducted alongside their paid labour. Specifically, many respondents discussed how conducting care work alongside domestic and paid labour added to their mental load. The weight of their mental load was compounded by the predominance of a ‘split’ or ‘never-ending’ shift and the presence or absence of support. Practical difficulties such as time and space were intertwined with the emotional labour the respondents conducted. These experiences were compounded by stress and strain, and for some respondents feeling isolated from support networks including their colleagues, familial networks, and more formal support such as creches, kindergartens, and schools.

Many of the narratives reflect how care obligations were added to the mental load working mothers carried during the pandemic, for example, by illustrating the toll of care on the weight of the respondent’s mental load. Care here includes emotional labour and the domestic labour involved in caring for their children and families. As noted in section two above, these behaviours and actions are connected with ‘maternal gatekeeping’ which argues that women take over the care and domestic duties as part of their performance of being an ‘ideal’ or ‘perfect’ mother. Taking over the bulk of domestic and care work in the home space connects with the feeling that they as mothers are best placed to do care and domestic tasks. Furthermore, it is connected with wanting to be a perfect mother. Undoubtedly, such pressure adds to the mental load of many working mothers, as is highlighted by the respondents who discussed never feeling that they were able to perform their mothering role sufficiently and how this negatively impacted their overall wellbeing and experience of mental load. Although the data used in this study refer specifically to the context of the pandemic, the narratives provide examples of issues that working mothers may encounter post-pandemic, such as the pressure of many working mothers endeavouring to be a perfect mother while engaging in paid work—trying to ‘do it all’—is not going to disappear post-pandemic.

It has been already noted that societal context impacted the experience of working mothers during the pandemic. However, while the narratives drawn from this study are from 11 countries with varying social, political, and welfare regimes, there are recurring common themes apparent in the data. As shown in the narratives here and in the wider literature reviewed, working mothers conducted increased levels of care work alongside their work during the pandemic. Moreover, increased care labour alongside paid labour impacted working mothers psychologically and in terms of their emotional wellbeing, adding to the mental load of this cohort of working mothers, thus revealing the strength of the nexus between care work and mental load.

The importance of support in relation to WfH while also juggling familial responsibilities was highlighted in the data, specifically how a lack of support may increase the negative side effects of carrying a heavy mental load. Many of the narratives demonstrate the difference in support (either its presence or its absence) made to the respondents’ mental load. For those parenting alone, the lack of support while caring for children and conducting paid labour added to their mental load. Indeed, while many women and mothers experienced increased mental load, this experience was more intense for those working mothers without strong support networks. For some of the respondents, having a supportive employer who made balancing paid and unpaid labour more manageable, the weight of their mental load was lessened. However, for those without support from family and/or from their employers, working and caring for their family members led to situations that impacted their mental health and led to feeling overwhelmed, as though they never did enough, and feelings of never being good enough. The salience of support regarding the experience of and the weight of the mental load is also illustrated in the narratives, particularly, in examples of how when conducting emotional labour as part of one’s paid employment resulted in exhaustion. This exhaustion with its knock-on impact on mental load was compounded for some respondents by a feeling that while they provided support for others that they did not have the same support lines open to them. Feeling and being supported makes a difference to the mental load a person carries. Thus, this is important for policymakers and researchers to be aware of, to factor into policy, and to be included on research agendas.

How the blurring of boundaries between one’s home space and workspace impacted the mental load carried by these respondents is highlighted in the data. For example, when the respondents discuss how having to work in shared spaces in the home such as kitchens caused friction within the family due to the perceived takeover of the home space. Home was no longer a space to escape the world of work. It actually became an ‘ambiguous space’ where care labour and paid labour were conducted in the same space which added to feeling overburdened. The narratives also showed how the idea of home not being a space to withdraw from paid work is connected with the ideas of both a ‘split shift’ and a ‘never-ending shift’. In the narratives, the respondents discuss fitting their unpaid work into their paid work. Blurred boundaries in relation to working mothers’ conducting split and never-ending shifts are illuminated by discussions of attending to care and domestic duties and then doing paid labour in what can be conceived as ‘break spaces’ from unpaid labour. Doing ‘everything’ in the same space added to the mental load these women carried and to the respondents’ feeling they had to do ‘everything’ at the expense of their personal lives. The respondents’ narratives suggest the importance of both physical space and mental space when WfH, and how the themes of care, support, and space are interconnected in relation to the experiences of these working mothers. Moving forward, the results discussed in this study raise questions in relation to working mothers who do not have the space for separate work spaces in their homes.

Finally, as noted in section three and section four, there are intersections visible thematically in the data. For example, some of the narratives illustrate how support and family status intersect and how support and care also intersect. The intersections that are illustrated in the narratives further reveal how the respondents’ mental load increased during the COVID-19 pandemic. Furthermore, the intersections go toward demonstrating how the mental load is affected by one’s social status and experiences in a fluid and dynamic manner.

## Limitations and future research

The RESISTIRÉ project aims to access individuals experiencing gender+ inequalities, and the national researchers who conducted the narrative interviews found ‘hard to reach’ groups difficult to access. An additional problematic aspect is that interview transcripts are turned into a shorter narrative text, which may lead to certain information being lost in the process.

Future research can adopt a more explicit gender+ approach, including further analysis on the unseen labour/mental load impacts on working mothers at the lower end of the labour market, as well as on the relationship between care obligations outside of childcare and mental load. For example, the experiences of working women and how care labour, for example, with elderly relatives impact their mental load and, subsequently, their wellbeing and careers. The results of this study could be strengthened by further research that includes a comparative analysis of the mental load during the COVID-19 pandemic as experienced by other groups—for example, women working from home without children and fathers working from home with children.

## Conclusion

There is a dearth of research using mental load theoretically to explore the connections and relationship between work and care on the mental load carried by working mothers. This study goes toward addressing this gap while contributing to the literature on mental load, working mothers, and WfH. Undoubtedly, the way many people work has been changed by the pandemic, and the move to WfH albeit in extenuating circumstances highlighted that many roles can be conducted at home. While the pandemic acted as a catalyst in relation to employers adopting flexible policies regarding employee presence in the workplace, WfH can lead to those working outside the office being side-lined and facing negative career impacts. There are also other potential negatives such as social isolation and reduced time spent doing paid labour to conduct unpaid domestic labour, which may have negative impacts on wellbeing and compound the negative impact on their career trajectories. This study provided a window into the experience of some working mothers during the pandemic. Areas that may provide social scaffolding for working mothers post-pandemic include the provision of support in relation to mental load, care, and paid labour. Employers and policymakers are important in creating workspaces—whether at home or in the employers’ space—that recognise the load carried by working mothers and support them in carrying it. In addition, it is important on a societal level to recognise mental load as salient and the negative impact it can have on all aspects of working mothers’ lives. Thus, the lessons from the pandemic need to be taken forward. One is the significance of state, societal, and social support for working mothers as illustrated in the data and the literature, and more specifically having work–family policies in place that support work–life balance. Another is the ability to set and maintain work–life balance boundaries (through having a ‘good’, supportive, well-paid job for example). These are all issues that have long been acknowledged as key factors in relation to wellbeing and career progression outcomes for working mothers. Reflecting on the findings presented above raises many questions about work and working mothers as WfH and hybrid work become entrenched practices in the world of work. Moving forward, it is necessary for policymakers to consider how the move toward increasingly hybrid work situations may impact working mothers in terms of both career and wellbeing outcomes. In essence, will the mental load get heavier as working mothers try to do everything while WfH and thereby reduce the time and mental space they have for paid work? This is arguably particularly pertinent for those who experience gender+ inequalities such as, women parenting alone who negotiate extra challenges in relation to work, wellbeing, and positive career trajectories and outcomes.

## Data availability statement

The processed data (narratives) supporting the conclusions of this article are available in Zenodo: https://doi.org/10.5281/zenodo.8347878 and https://doi.org/10.5281/zenodo.8345018.

## Author contributions

CD conceived the idea for the study and wrote the overall document. SC edited the document. AB and SC contributed to the literature review and presentation of data. All authors contributed to the article and approved the submitted version.
